# Alpha-synuclein seeding activity in *postmortem* tissues from patients with diffuse and isolated Lewy bodies

**DOI:** 10.1186/s40478-025-02195-6

**Published:** 2026-01-07

**Authors:** Soňa Baranová, Radoslav Matěj, Jakub Soukup, Petr Dušek, Karel Holada

**Affiliations:** 1 Institute of Medical Microbiology, First Faculty of Medicine, Charles University, Prague, Czech Republic; 2 Department of Pathology and Molecular Medicine, Third Faculty of Medicine, Charles University and Thomayer University Hospital, Prague, Czech Republic; 3 Department of Pathology, Third Faculty of Medicine, Charles University and University Hospital Kralovske Vinohrady, Prague, Czech Republic; 4 Department of Pathology, First Faculty of Medicine, Charles University and General University Hospital, Prague, Czech Republic; 5 Department of Neurology, Center of Clinical Neuroscience of the First Faculty of Medicine, Charles University and General University Hospital, Prague, Czech Republic

**Keywords:** Alpha-synuclein, Synucleinopathy, Comorbidities, Seeding amplification assay, SAA, Real-time quaking-induced conversion assay, RT-QuIC, Seeding activity

## Abstract

We have evaluated the diagnostic potential of the seeding amplification assay (SAA) in detecting α-synuclein seeding activity in *postmortem* brain and cerebrospinal fluid (CSF) samples from patients with primary and co-pathology α-synucleinopathies. Moreover, we investigated potential SAA positivity in control samples which may suggest unrecognized co-pathology. A total of 15 brain and 14 CSF samples with definite dementia with Lewy bodies (DLB, n = 6), Alzheimer´s disease with amygdala Lewy body (AD/ALB, n = 3), and patients with concomitant Creutzfeldt-Jakob disease and Lewy body pathology (CJD/LBP, n = 6) comorbidity were tested for α-synuclein seeding activity using SAA assay utilizing recombinant α-synuclein (WT) with N-terminal His-tag. Control samples consisted of other neurodegenerative diseases (n = 17 for brain and n = 18 for CSF samples) and healthy corneal donors (n = 17). The analysis of seeding activity in brain samples suggested 100% sensitivity and 91.2% specificity. Five out of 34 brain control samples gave a positive SAA outcome. However, upon reevaluation, two of these samples were reclassified as Alzheimer´s disease (AD) with synucleinopathy co-pathology. The analysis of CSF also suggested 100% sensitivity and 94.4% specificity, although dilution of some samples was necessary to decrease the effect of inhibitors. We report a good performance of the SAA not only in *postmortem* samples from primary synucleinopathies with advanced pathology, but also in co-pathology synucleinopathies with isolated Lewy bodies in the amygdala in AD cases. Our findings highlight the importance of careful diagnostic evaluation in AD patients, where co-existing synucleinopathy may otherwise go unrecognized.

**Supplementary Information:**

The online version contains supplementary material available at 10.1186/s40478-025-02195-6.

## Introduction

Synucleinopathies represent a group of progressive neurodegenerative diseases characterized by the accumulation of pathological alpha-synuclein (ɑ-syn^D^) in the central and peripheral nervous system. Deposits of aggregated ɑ-syn^D^ can be found *postmortem* in brain either within neurons as the main component of Lewy bodies (LB) which is characteristics for Parkinson´s disease (PD), Parkinson´s disease dementia (PDD) and dementia with Lewy bodies (DLB) or as glial cytoplasmic inclusions in multiple system atrophy (MSA) known as Papp-Lantos bodies [43, 45]. Lewy body dementias account for 30% of all dementia cases and are ranked as the second most common form of neurodegenerative dementias, after Alzheimer´s disease (AD) [13]. Despite its high prevalence, clinical diagnosis is based mostly on the patient’s history and the evaluation of motor and non-motor symptoms. However, clinical symptoms of neurodegenerative disorders often overlap, leading to the possibility of misdiagnosis [12]. At present, a definite diagnosis can be made only *postmortem* by neuropathological examination.

In the pursuit of a specific *antemortem* diagnosis, the application of Seeding amplification assay (SAA), specifically Real-Time Quaking-Induced Conversion (RT-QuIC) for synucleinopathies, was proposed. SAA was originally developed as an ultrasensitive and specific diagnostic tool for prion diseases using cerebrospinal fluid (CSF) [2], olfactory mucosa [29], or skin [31] samples. However, in the last years, the assay was adapted for α-synucleinopathies utilizing the ‘prion-like’ seeding activity of α-syn^D^ in patients’ CSF samples, achieving high diagnostic accuracy [14]. Rossi et al. reported 95.2% sensitivity and 98% specificity in neuropathologically confirmed LB cases. In the same study, Rossi et al. confirmed 97.1% sensitivity and 86.7% specificity in living patients with a clinical diagnosis of dementia [35]. Furthermore, in the blind comparative study, the 86% sensitivity and 97% specificity were reached using CSF of PD patients [38]. In a cross-laboratory study, four laboratories demonstrated a high level of agreement exploiting two protocols adapted from Parchi’s group at ISNB in Bologna and Amprion Inc [9]. On the other hand, in MSA CSF samples, sensitivity varies from 4.4 to 75% while the specificity ranges from 66.7 to 100% [19]. In the last years, it has become clear that the existence of mixed pathologies in neurodegenerative patients is more common than previously thought, posing challenges for accurate diagnosis [33, 43].

One of the many challenges in clinical diagnosis is the classification of neurodegenerative diseases primarily based on the predominant proteinopathy. For instance, in 20–50% of AD cases, LB pathology is observed [1, 23]. In a small subset of these patients, LB are localized only in the amygdala. This gives rise to a specific subtype known as amygdala-restricted Lewy body (ALB) pathology, which accounts for 6% of AD/LB comorbidity [39]. Similarly, tau and Aβ pathology can be commonly found in Creutzfeldt–Jakob disease (CJD) brains [32]. In the Czech Republic, 62% of patients diagnosed with CJD displayed comorbidity with some type of tauopathy [21]. Other CJD co-pathologies, such as α-synucleinopathies or TAR DNA-binding protein 43 (TDP-43) proteinopathy, were also reported but are comparatively rare [21, 37]. Still, only a limited number of studies analyzed samples with α-synucleinopathy co-pathologies, and it is not clear how well SAA applies for them [7, 41, 42].

In the present study, we aimed to evaluate the presence of seeding activity of α-syn^D^ in archived brain homogenate (BH) and CSF samples obtained from patients with neuropathologically confirmed primary α-synucleinopathy (DLB), as well as from patients with co-pathology α-synucleinopathy, such as Alzheimer’s disease with amygdala Lewy body (AD/ALB), and patients with concomitant Creutzfeldt-Jakob disease and Lewy body pathology (CJD/LBP).

## Patients and methods

### Patients’ samples

The cohort of patients´ samples analyzed in our retrospective study was provided by the National Reference Laboratory for Human Prion Diseases in Prague. This study was approved by the ethical committee of the Institute for Clinical and Experimental Medicine and Thomayer University Hospital in Prague (G-22-27). In total, 15 standardized BH were prepared from left frontal lobe and 14 ventricular cerebrospinal fluid (CSF) samples were obtained from patients with a definite diagnosis of synucleinopathy between 2012 and 2021 as described previously [3, 28] and stored frozen at − 80 °C. The CSF sample from one patient with CJD/LBP was not available for the analysis. Definite diagnoses of synucleinopathies and other neurodegenerative diseases were confirmed neuropathologically by immunohistochemistry during the autopsy.

The control group for BH samples consisted of patients with other neurodegenerative diseases (OND, n = 17) as well as anonymized healthy corneal donors (CD, n = 17). The control group for ventricular CSF samples consisted of distinct patients with other neurodegenerative diseases (n = 18).

### Neuropathological evaluation of brain samples

Brains were fixed for 3–4 weeks in buffered 10% formalin, and the selected tissue blocks were embedded into paraffin (FFPE) following a standardized protocol [34]. 5-µm-thick sections were stained with hematoxylin-eosin, Klüver-Barrera, and silver impregnation methods.

Deparaffinized 5-µm-thick FFPE tissue sections were incubated (20 min, RT) with primary antibodies. Treatment with 96% formic acid was applied prior to β-amyloid and prion protein staining. Mouse monoclonal antibodies: prion protein-12F10 (1:8000; Bertin Pharma #A03221) and 6H4 (1:3000; Prionics #7500996), Amyloid ß-6 F/3D (1:1000; Dako #M0872), Phospho-Tau-AT8 (1:500; Thermo Fisher Scientific #MN1020), Phospho TDP-43-11-9 (1:4000; Cosmo Bio #TIP-PTD-M01), Alpha-Synuclein-5G4 (1:1000; Dianova #NDG-76506) and. EnVision FLEX/HRP system (Dako #M822) was utilized for visualization using DAB substrate.

The labeled sections were counterstained with Mayer’s Hematoxylin Solution. AD was scored using the National Institute on Aging–Alzheimer’s Association consensus scheme [20] and DLB using the consensus criteria [27] with a determination of the Braak stage [8].

### Purification of recombinant α-synuclein

Recombinant wild-type human α-synuclein with N-terminal HisTag (rec.αSyn) was prepared as published [16]. Briefly, the overexpression of rec.ɑSyn in bacterial cells was induced with Overnight Express Autoinduction System 1 (Novagen). The next day, the cells were treated with osmotic shock buffer (40% sucrose, 30 mM Tris-HCl, 2 mM EDTA, pH 7.2) and ice-cold milli-Q water (mQH_2_O) with 5 mM MgCl_2_ to release the protein from the periplasmic space. Contaminating proteins were precipitated by lowering the pH to 3.5 and sedimented by centrifugation. The supernatant adjusted to pH 7.5 was filtered through 0.22 μm syringe filter, loaded onto HisTrap Fast Flow (Cytiva) 5 ml column and eluted with linear gradient of imidazole. The peak containing rec.αSyn was collected and loaded onto a HiTrap Q-HP 5 ml column (Cytiva) and eluted with a linear gradient of NaCl. The purified protein was dialyzed overnight at 4 °C against water or 40 mM phosphate buffer, pH 8.0 using 3 kDa MWCO SnakeSkin™ dialysis membrane (Thermo Scientific). The absorbance at 280 nm was measured, and the concentration of the protein was calculated with an extinction coefficient ε_280_^0.1%^ = 0.36. The aliquots of rec.ɑSyn (1 mg/ml) were stored frozen at − 80 °C.

Recombinant shortened hamster prion protein (rec. HaPrP90-230) was prepared as previously described [28].

### Analysis of α-synuclein seeding activity of patients’ samples by SAA

The seeding activity was detected by SAA, specifically RT-QuIC, described by Groveman et al. [16], with minor modifications. Archival frontal cortex 10% BHs in buffer containing 0.5% sodium deoxycholate and 0.5% Tergitol NP-45 were prepared and stored frozen as previously described [28]. The BH samples were analyzed in the presence or absence of sodium dodecyl sulfate (SDS) and N-2 supplement.

Brain samples were analyzed as BH in final dilutions from 10^− 2^ up to 10^− 8^. Initially, BHs were diluted and analyzed only in PBS, pH 7.4. Subsequently, to improve the performance of the assay, BHs were diluted and analyzed in a buffer containing PBS, pH 7.4, 0.025% SDS, and 1x N-2 supplement. The reaction mixture was composed of 40 mM phosphate buffer (pH 8.0), 170 mM NaCl, 10 µM ThT, and 0.1 mg/ml rec.αSyn. 2 µl of the diluted sample was added into 98 µl of the reaction mix in the well of non-treated black 96-well plate with optical bottom (cat. No. 2102.0372, Nunc, Fisher Scientific) supplemented with six 0.8-micron silica beads (OPS Diagnostics).

For CSF samples, 15 µl of undiluted or 10x diluted CSF in PBS was added into 85 µl of the reaction mix containing 40 mM phosphate buffer (pH 8.0), 170 mM NaCl, 10 µM ThT, and 0.1 mg/ml rec.αSyn with 0.0015% SDS.

All samples were analyzed in quadruplicate. The plate was incubated in the FLUOstar Omega fluorescence reader (BMG LABTECH GmbH, Germany) at 42 °C with repeating cycles of rest (1 min) and double-orbital shaking (400 rpm, 1 min). BH samples diluted in PBS and CSF samples were incubated for 60 h, whereas BH samples containing SDS were incubated for 48 h. The fluorescence was read every 15 min at 450 ± 10 nm excitation and 480 ± 10 nm emission.

Both brain and CSF samples of patients with CJD/LBP comorbidity were also tested by prion SAA, the second-generation RT-QuIC, as previously described [28]. Brain samples were analyzed as BH following final dilutions of brain tissue from 5 × 10^− 7^ to 5 × 10^− 11^, except for one sample, which required further dilution to 5 × 10^− 13^. CSF samples were analyzed either undiluted or 10x diluted. Briefly, 2 µl of BH or 15 µl of CSF was added into a reaction mix, containing 11.9 mM phosphate, 1 mM EDTA, 310 mM NaCl (pH 7.4), 10 µM ThT, and 0.1 mg/ml recombinant hamster prion protein (rHaPrP90-231) into a final volume of 100 µl. For CSF, the reaction mix was, in addition, supplemented with 0.002% SDS. Samples were incubated for 48 h at 55 °C with repeating 1 min cycles of intermittent shaking (700 rpm) and 1 min incubation.

### Analysis of SAA data

For each sample, the mean from all four wells was calculated and used in the subsequent SAA analysis. The outcome of the α-syn assay was determined by the threshold calculated for brain and CSF samples separately. For the brain, the threshold was calculated from the signal of the control group of corneal donors (CD), and for CSF samples from the group of other neurological controls (OND). The threshold values were expressed as the mean max ThT fluorescence + ten times the standard deviation (SD) using the mean max ThT values of control samples within 1 SD. The threshold calculations did not include the control samples with values above the SD. Furthermore, one CSF sample with hypoxic/anoxic brain injury was omitted from calculations due to a high background signal. Samples were classified as positive when the mean max ThT fluorescence from four wells exceeded the threshold and at least two of the four wells showed an increase in fluorescence signal above the baseline. For prion SAA, threshold values were calculated previously [28]. The mean time to threshold was determined using values obtained for each sample, excluding the ones that did not reach the threshold.

For both α-syn and prion assays, the seeding dose 50 (SD_50_) was determined as previously described [44]. Briefly, an analogous Spearman-Kärber analysis was used to determine the dilutions at which two wells exhibited positive seeding activity. The last dilution with four positive wells and the first dilution with zero positive wells were used for the calculations using formula: ${x}_{p=1}+\frac{1}{2}d-d\sum_{{x}_{p=1}}^{{x}_{min}}{p}_{x}$.

### Determination of SAA analytical sensitivity

The analytical sensitivity of α-syn SAA was verified by seeding with pre-formed rec.αSyn fibrils (PFFs) in end-point dilution. The PFFs were sonicated for 30 s and serially diluted in a 10x diluted negative control CSF. The SAA reaction was performed as for the CSF samples (Supplemental data).

### Statistical analysis

The results from SAA were plotted and statistically analyzed using GraphPad Prism 5 (GraphPad Software Inc.). Data normality was assessed using the Shapiro–Wilk test. Comparisons between groups were performed using an unpaired *t*-test for normally distributed data or the Mann–Whitney test for non-normal data distribution. Differences were considered statistically significant at *P* < 0.05. The Student’s *t*-distribution confidence interval (CI) for the mean and the binomial proportion CI using the Clopper–Pearson exact method were calculated using Microsoft Excel.

## Results

### Patients’ cohort

All tested samples had a definite diagnosis confirmed by neuropathological evaluation at autopsy. The diagnoses included dementia with Lewy bodies (DLB, n = 6), Alzheimer’s disease with amygdala Lewy body (AD/ALB, n = 3), and patients with concomitant Creutzfeldt-Jakob disease and LBP (CJD/LBP, n = 6) (Table 1). Detailed characteristics of patients from both the brain and CSF neurological control groups are provided in Supplementary Tables 1 and 2.

**Table 1:** Characteristic, demographic information, and the SAA positivity of the cohort of patients with synucleinopathy

Patient	Age	Sex	PMI [h]/storage time [years]	Definite diagnosis	Braak staging	McKeith staging	SAA result for brain	SAA results for CSF dilution 10^0^/10^− 1^
01	83	M	NA/5	DLB	4	Limbic/transitional	+	+/+
02	71	M	80/1	DLB	6	Diffuse neocortical	+	+/+
03	78	M	35/1	DLB	6	Diffuse neocortical	+	+/+
04	74	F	39/1	DLB	6	Diffuse neocortical	+	+/+
05	88	F	22/1	DLB	5	Limbic/transitional	+	+/+
06	87	M	87/1	DLB	5	Diffuse neocortical	+	+/+
07	97	F	72/3	AD (A3B3C3) / ALB	–	Amygdala-predominant	+	+/+
08	84	F	87/2	AD (A3B3C3) / ALB	–	Amygdala-predominant	+	+/+
09	80	M	48/1	AD (A3B3C3) / ALB	–	Amygdala-predominant	+	+/−
10	60	F	34/8	CJD/LBP	3	Brainstem predominant	+	+/+
11	71	F	110/8	CJD/LBP	3	Brainstem predominant	+	N/A
12	77	M	30/2	CJD/LBP	4	Limbic/transitional	+	+/−
13	70	F	26/2	CJD/LBP	6	Diffuse neocortical	+	−/+
14	72	M	56/2	CJD/LBP	4	Limbic/transitional	+	+/+
15	83	F	23/1	CJD/LBP	6	Diffuse neocortical	+	+/+

Patients positive for dementia with Lewy body depositions were classified according to Braak and McKeith staging system. Brain homogenates were analyzed by SAA protocols with or without the presence of SDS. Cerebrospinal fluid samples were analyzed either undiluted or 10x diluted

DLB—Dementia with Lewy bodies, AD/ALB—Alzheimer’s disease/Amygdala Lewy body comorbidity, CJD/LBP—concomitant Creutzfeldt-Jakob disease and Lewy body pathology, PMI—*postmortem* interval, SDS—sodium dodecyl sulfate, BH—brain homogenate, CSF—cerebrospinal fluid, SAA—Seeding amplification assay

### Detection of alpha synuclein positivity in brain tissue samples by immunohistochemistry

**Fig. 1:**
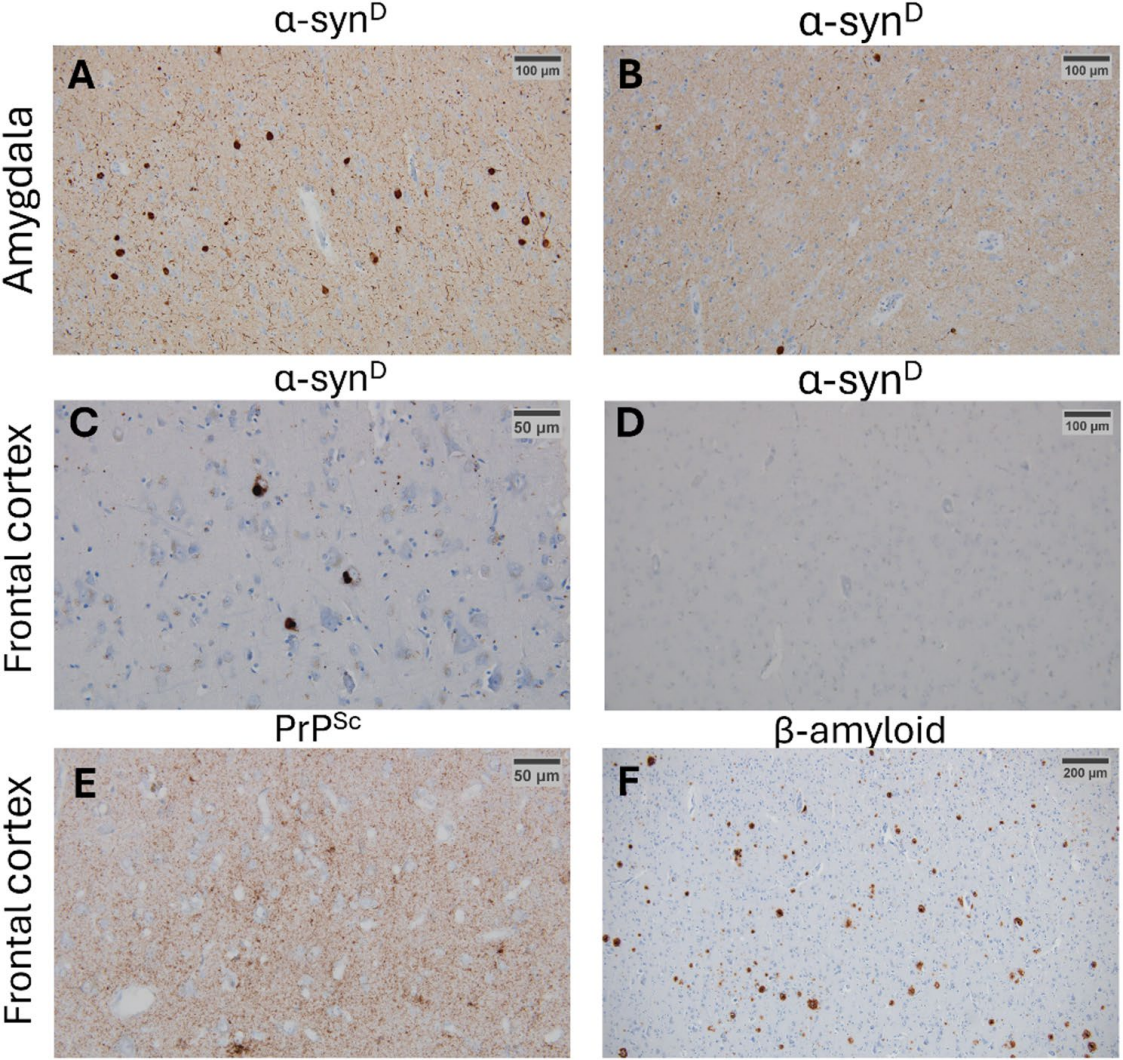
Immunohistochemical staining of pathological α-synuclein, prion and β-amyloid deposits in different areas of the brain. Differences in density of Lewy bodies in amygdala from patient with DLB (**A**) and AD/ALB comorbidity (**B**). Detection of a-syn^D^ deposits in frontal cortex from patient with DLB (**C**) and lack thereof in AD/ALB comorbidity (**D**). Presence of pathological prion protein PrP^Sc^ (**E**) and β-amyloid (**F**) deposits in the frontal cortex from patients with CJD/LBP and AD/ALB, respectively. DLB—Dementia with Lewy bodies, AD/ALB—Alzheimer’s disease/Amygdala Lewy body comorbidity, CJD/LBP—concomitant Creutzfeldt–Jakob disease and Lewy body pathology, PrP^Sc^—pathological prion protein

All DLB and CJD/LBP cases, revealed the α-syn^D^ positivity in the samples of amygdala (Fig. 1A) and frontal cortex (Fig. 1C). In the AD/ALB cases, the α-syn^D^ positivity was detected only in amygdala (Fig. 1B), and the cortex was devoid of any immunohistochemical reactions (Fig. 1D). Similarly, the analysis of control OND samples revealed negative α-syn^D^ reactions of all immunohistochemically stained samples from the frontal cortex (not shown). Frontal cortex of CJD/LBP and AD/ALB cases was positive for pathological prion protein (PrP^Sc^) and β-amyloid staining, respectively (Fig. 1E, F).

### Evaluation of SAA ability to detect rec.αSyn pre-formed fibrils

The successful formation of rec.αSyn PFFs was confirmed by ThT assay and by the visualization of amyloid fibrils by TEM imaging (Supplementary Fig. 1A, B). SAA analysis of serially diluted PFFs demonstrated the presence of seeding activity up to 10 pg/ml of rec.αSyn PFFs suspension (Supplementary Fig. 1C).

### Detection of the α-syn^D^ seeding activity in brain samples using the SAA protocol without SDS

**Fig. 2:**
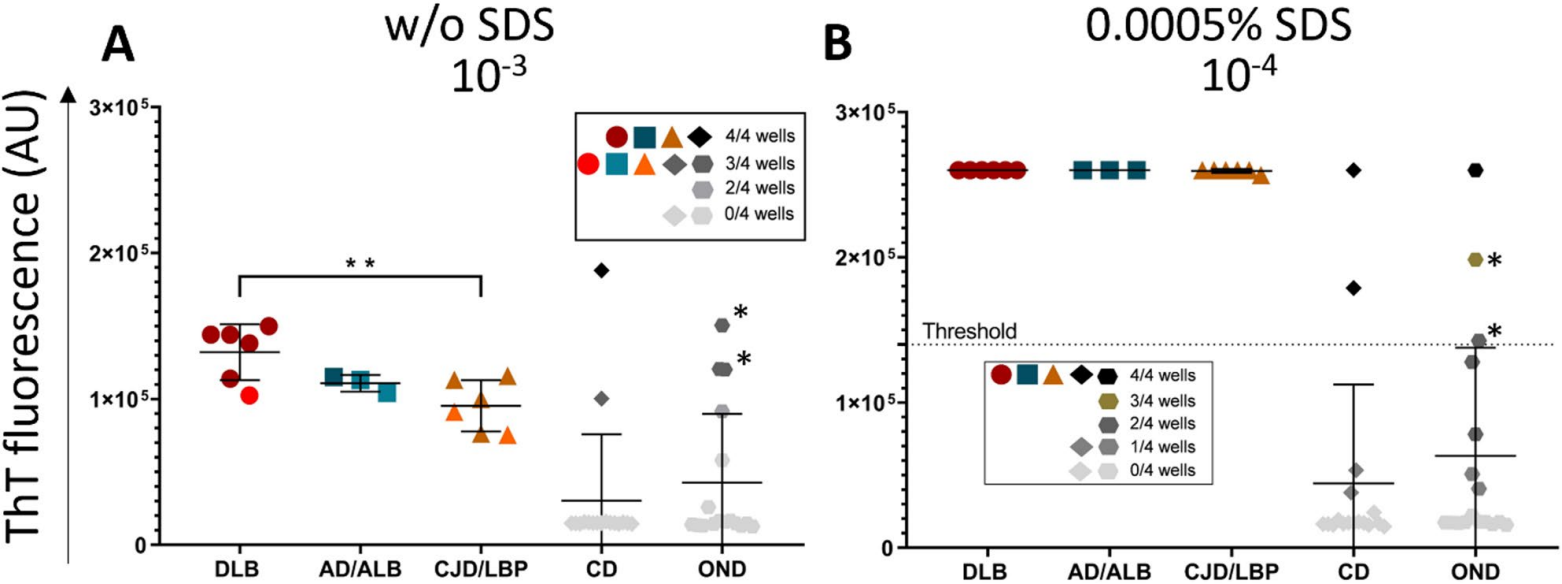
Comparison of maximal ThT fluorescence intensity of individual brain samples analyzed by SAA. Samples were analyzed in quadruplicates and the mean max fluorescence of four wells was plotted in the graph for every individual. Dots with asterisks represents two AD patients that were re-classified after the immunohistochemical reanalysis. Horizontal lines show the mean ± standard deviation (SD). The number of wells displaying seeding activity is represented by different color shades. **A** The max ThT signal of brain samples tested in the absence of SDS at the dilution of 10^− 3^. **B** The max ThT signal of brain samples tested in the presence of SDS at the dilution of 10^− 4^. Samples were considered positive when the mean max ThT signal exceeded the calculated threshold (dashed line). **P < 0.005, AU—arbitrary fluorescence unit. DLB—dementia with Lewy bodies (n = 6), AD/ALB—Alzheimer’s disease/Amygdala Lewy body comorbidity (n = 3), CJD/LBP—concomitant Creutzfeldt–Jakob disease and Lewy body pathology (n = 6), CD—corneal donors (n = 17), OND—other neurodegenerative diseases (n = 17), SAA—seeding amplification assay

**Fig. 3:**
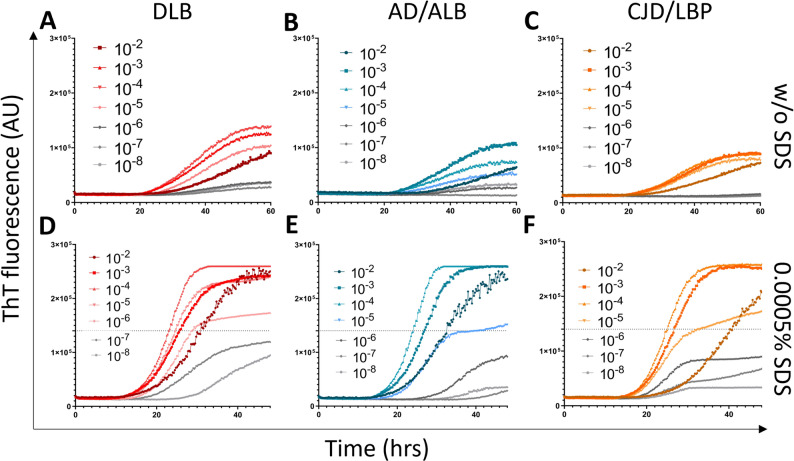
SAA end-point dilution analysis of α-syn^D^ seeding activity in brain tested in the absence or presence of SDS. Samples were serially diluted (10^− 2^ to 10^− 8^) and tested in quadruplicates. Each dilution trace represents the mean ThT fluorescence of all tested samples at given timepoint. **A**–**C** The analysis of the α-syn^D^ seeding activity in the absence of SDS, test duration 60 h. **D**–**F** The enhancement of the mean fluorescence response of α-syn^D^ analyzed in the presence of SDS, test duration 48 h. Dashed line indicates the threshold of the assay for the positive SAA result at 10^− 4^ dilution. The signal of least diluted brain samples is partially quenched. AU—Arbitrary fluorescence unit, DLB—dementia with Lewy bodies (A + D, n = 6, red), AD/ALB—Alzheimer’s disease/Amygdala Lewy body comorbidity (B + E, n = 3, blue), CJD/LBP—concomitant Creutzfeldt–Jakob disease and Lewy body pathology (C + F, n = 6, orange), SAA—seeding amplification assay

The archive BHs prepared from the frontal cortex (n = 49) were analyzed using two different SAA conditions. First, the serial dilutions (10^− 2^ to 10^− 8^) of brain in PBS (pH 7.4) were analyzed using rec.αSyn substrate dialyzed against mQH_2_O and no SDS was added to the reaction mixture. All samples with definite synucleinopathy (n = 15) gave a positive ThT fluorescence signal (Fig. 2A). In general, the positive signal was detected up to 10^− 5^ dilution. DLB (n = 6) cases gave the highest average ThT signal when diluted 10^− 4^, whereas AD/ALB (n = 3) and CJD/LBP (n = 6) cases at 10^− 3^ dilution (Fig. 3A–C). The dispersion of ThT values was lowest at 10^− 3^ dilution (Supplementary Fig. 2).

At 10^− 3^ dilution, DLB and AD/ALB samples had mean max ThT fluorescence 13 ± 1.9 × 10^4^ and 11 ± 0.6 × 10^4^, respectively (Fig. 2A). The mean fluorescence of CJD/LBP samples was significantly lower (9.5 ± 1.8 × 10^4^, *P* < 0.01) than that of DLB cases. Area under the curve (AUC) for DLB, AD/ALB, and CJD/LBP samples was 3.4 × 10^6^, 2.9 × 10^6^ and 2.6 × 10^6^, respectively (Fig. 3A–C). The highest mean SD_50_ in brain tissue, estimated using tenfold endpoint dilution, was 10^7.7^/g (95% CI 10^6.65^, 10^8.78^) for DLB cases. For AD/ALB and CJD/LBP cases, the mean SD_50_ was 10^6.8^/g (95% CI 10^5.34^, 10^8.33^) and 10^7.5^/g (95% CI 10^6.8^, 10^8.16^), respectively (Table 2). All values for individual samples are shown in Supplementary Table 3.

**Fig. 4:**
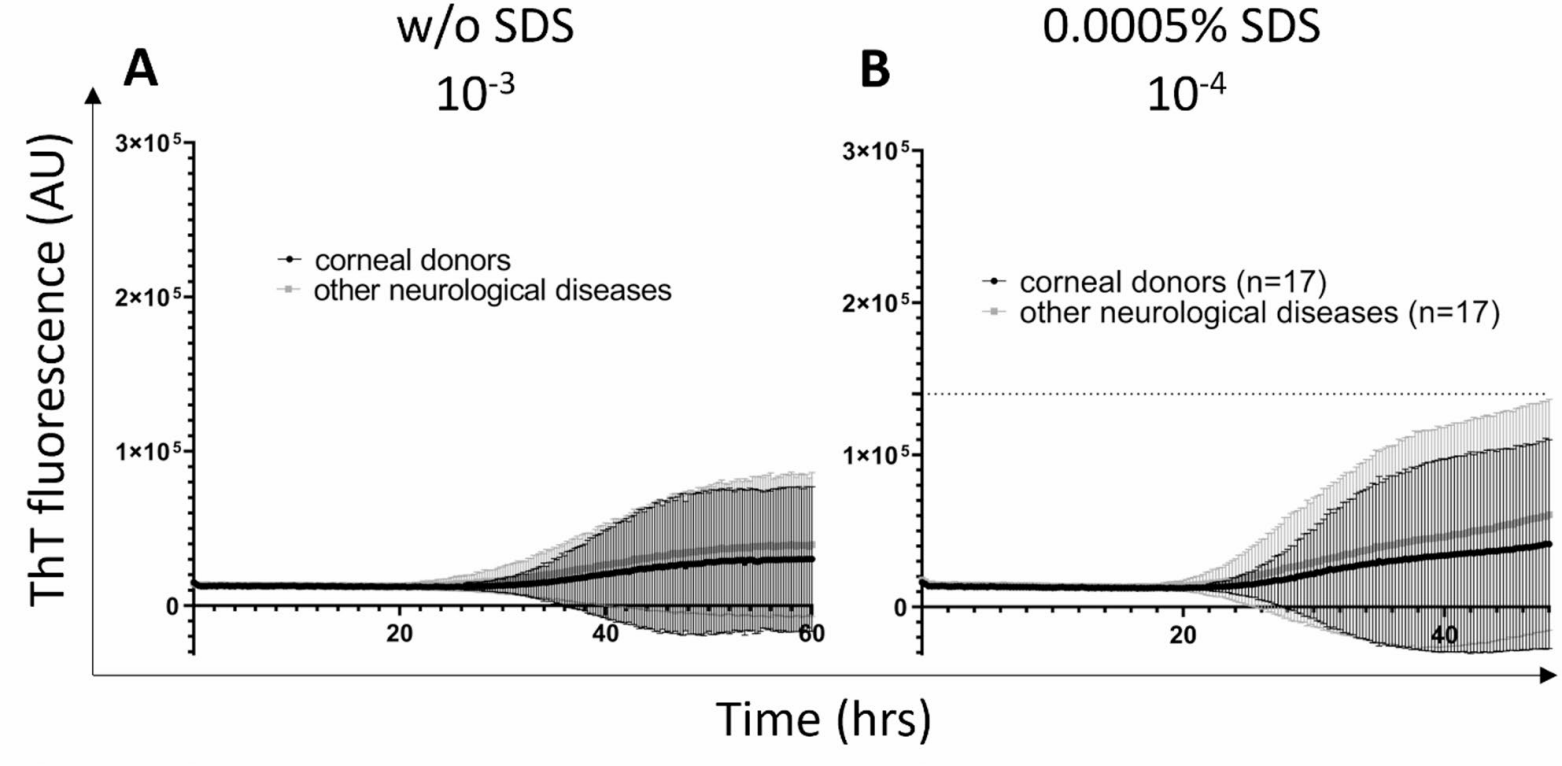
SAA analysis of α-syn^D^ seeding activity in control brain samples at the appropriate sample dilution in the absence or presence of SDS. Controls samples of corneal donors (n = 17, black) and other neurological diseases (n = 17, grey) were analyzed in quadruplicates. Each trace represents the mean ThT fluorescence ± standard deviation (SD) of all tested samples at given timepoint. **A** The results of SAA analysis of brain samples diluted 10^− 3^ in the absence of SDS. Test duration 60 h. **B** The results of analysis of control samples diluted 10^− 4^ in the presence of SDS. Test duration 48 h. Dashed line indicates the calculated threshold for the positive SAA results. AU—Arbitrary fluorescence units, SAA—seeding amplification assay

Five out of 34 controls, including two corneal donors (CD) and three Alzheimer’s patients (AD) displayed the ThT signal above the standard deviation of their respective group and it overlapped with the signal of synucleinopathy samples (Fig. 2A). The mean max ThT fluorescence of all CD samples (n = 17) at 10^− 3^ dilution was 3 ± 4.6 × 10^4^ and of all OND samples (n = 17) 4 ± 4.7 × 10^4^ (Fig. 4A). Mean AUC for all CD samples was 1.1 × 10^6^ and for all OND cases 1.3 × 10^6^ (Fig. 4A).

### Reevaluation of SAA positive control brain samples by immunohistochemistry

To confirm the absence of α-syn^D^ brain deposits in the SAA positive OND cases (n = 3, all AD), the FFPE archive tissues were reanalyzed by immunohistochemistry, and different areas of the brain were inspected. Indeed, α-syn^D^ deposits were observed in the amygdala of one AD patient thus demonstrating neuropathological changes compatible with AD/ALB. The second AD case revealed the α-syn^D^ deposits in amygdala, cingular cortex, hippocampal region, and mesencephalic structures corresponding to the limbic transitional stadium of LBP and corresponding to AD/LBP co-pathology. The third reevaluated AD case was α-syn^D^ immunohistochemistry negative. Corneal donor samples were excluded from re-examination as FFPE tissue was not available. Exclusion of the comorbidity AD samples (n = 2) lowered the mean max ThT fluorescence of OND (n = 15) samples to 3 ± 3.3 × 10^4^ and AUC to 1.1 × 10^6^.

### Detection of the α-syn^D^ seeding activity in the brain using a modified SAA protocol with SDS improves the assay performance

Due to the low overall ThT fluorescence, we introduced two minor modifications to enhance the SAA signal. Brain samples were analyzed in end-point dilutions (10^− 2^ to 10^− 8^), using PBS with 0.025% SDS and N-2 supplement. The rec.ɑSyn substrate was dialyzed against 40 mM PB (pH 8.0).

All brain samples with definite synucleinopathy (n = 15) gave a positive ThT signal, suggesting 100% (95% CI 78.2–100%) assay sensitivity. The max fluorescence was noticeably higher, in nearly all cases hitting the detection limit (~ 26 × 10^4^ AU) (Fig. 2B). DLB cases gave positive ThT signal even when diluted 10^− 6^, whereas AD/ALB and CJD/LBP comorbidities up to 10^− 5^ dilutions (Fig. 3D–F). The highest mean ThT max values were achieved at 10^− 4^ dilution (Supplementary Fig. 3).

The mean max ThT fluorescence at 10^− 4^ dilution was the same (26 × 10^4^) for DLB, AD/ALB, and CJD/LBP samples (Fig. 2B), representing the detection limit of the measurement. Mean time to threshold (TTT) was 23 ± 2 h for DLB cases, 24 ± 1 h for AD/ALB, and 25 ± 3 h for CJD/LBP. The AUC was 7 × 10^6^ in DLB cases, while the AUC for AD/ALB and CJD/LBP cases was 6.7 × 10^6^ and 6.3 × 10^6^ (Fig. 3D–F). The mean SD_50_ was estimated for all samples except one DLB (Braak 6, McKeith´s diffuse neocortical stage) case, where the seeding activity was detected in all four wells even at 10^− 8^ dilution (Supplementary Table 4). The calculated values of mean SD_50_ were similar for all groups of patients, 10^9.3^/g (95% CI 10^8.01^, 10^10.63^), 10^8.6^/g (95% CI 10^7.09^, 10^10.11^), and 10^8^/g (95% CI 10^5.88^, 10^10.12^) in DLB, CJD/LBP and, AD/ALB, respectively (Table 2).

Again, out of 34 controls, the same five samples, two CD and three ADs, were classified as positive (Fig. 2B), suggesting 85.3% (95% CI 68.9–95%) assay specificity. Subtraction of the two AD patients reanalyzed as AD/ALB and AD/LBP improved the specificity to 91.2%. The mean max ThT fluorescence for CD (n = 17) was 4.4 ± 6.8 × 10^4^ and for OND (n = 17) was 6.3 ± 7.5 × 10^4^ (Fig. 4B). Exclusion of the two α-syn^D^ positive AD cases lowered the mean max ThT fluorescence of OND (n = 15) to 4.9 ± 6.6 × 10^4^. The time to threshold for one false positive AD case was 29 h, and for the reanalyzed AD/ALB and AD/LBP cases was 37 and 41 h, respectively. For the two positive CD samples, it was 31 and 40.75 h. The mean AUC of brain control samples was comparable to the first setup, with 1 × 10^6^ for CD samples and 1.3 × 10^6^ for OND samples (Fig. 4B), which lowered to 0.6 × 10^6^ omitting the re-classified AD α-syn^D^ positive cases (n = 2).

**Table 2:** Mean seeding dose of 50% (SD_50_) in brain from patients with confirmed synucleinopathies determined with SAA assay

	w/o SDS		0.0005% SDS	
	log_10_ SD_50_/2 µl	log_10_ SD_50_/1 g	log_10_ SD_50_/2 µl	log_10_ SD_50_/1 g
DLB (n = 6)	5	7.7	6.6*	9.3*
AD/ALB (n = 3)	4.2	6.8	5.3	8
CJD/LBP (n = 6)	4.8	7.5	6	8.6

Samples were analyzed serially ten-fold diluted up to 10^− 8^

DLB—Dementia with Lewy bodies, AD/ALB—Alzheimer’s disease/Amygdala Lewy body comorbidity, CJD/LBP—concomitant Creutzfeldt–Jakob disease and Lewy body pathology, SAA—seeding amplification assay

*SD_50_ for sample no. 4 (DLB, Braak stage 6/McKeith´s diffuse neocortical stage) could not be determined as the samples showed the seeding activity in all four wells even in the highest dilution, therefore the dilution 10^− 8^ was used in the calculations

### Detection of α-syn^D^ seeding activity in postmortem cerebrospinal fluid

**Fig. 5:**
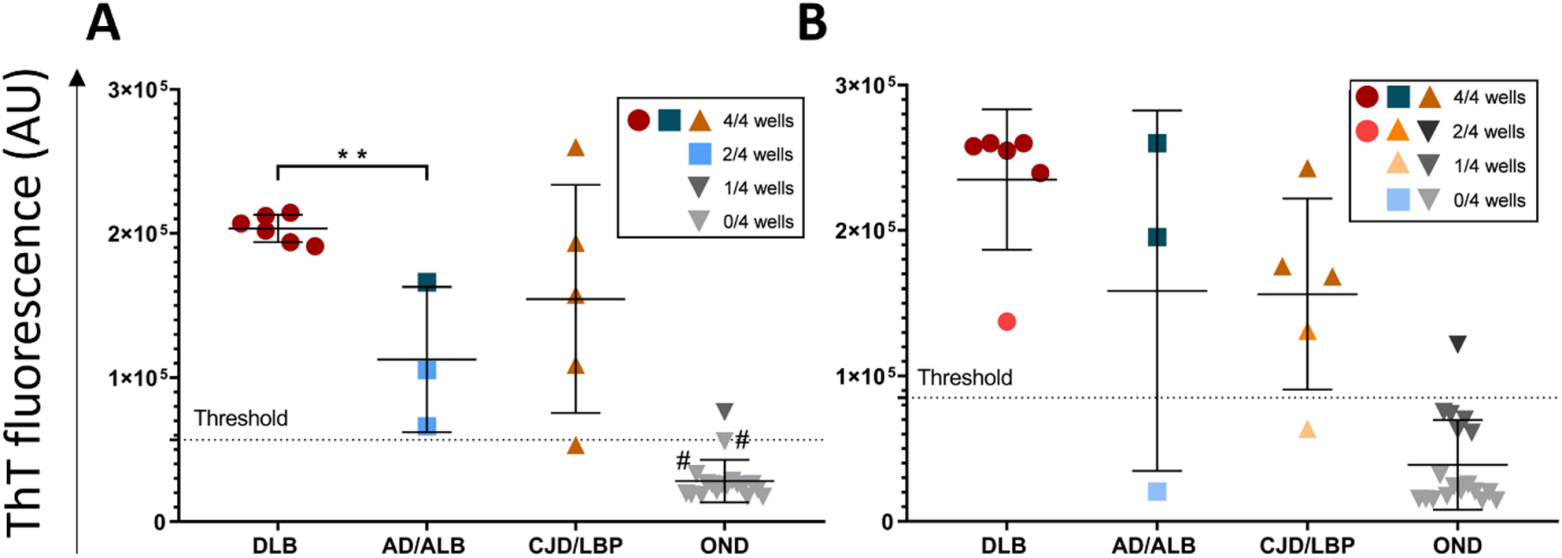
Comparison of maximal ThT fluorescence intensity of individual *postmortem* CSF samples analyzed by α-syn^D^ SAA. The analysis of undiluted (**A**) and 10x diluted (**B**) CSF samples of DLB (n = 6) AD/ALB (n = 3), CJD/LBP (n = 5) and control OND (n = 18) cases. The samples were analyzed in quadruplicates and the mean max ThT fluorescence for each sample was plotted in the graph. The number of wells displaying seeding activity is represented by different color shades. Samples were considered positive when the max ThT signal exceeded the threshold (dashed line). Every dot represents the mean max ThT fluorescence of four wells of the tested sample. Horizontal lines show the mean ± standard deviation (SD). One undiluted AD and one hypoxic/anoxic brain injury sample (#) showed unusual high baseline signal from the beginning of measurement, but without any increase, so the values of ThT fluorescence at the end of the assay were used in the graph and the samples were omitted from threshold calculations. **P < 0.005, AU—arbitrary fluorescence unit. DLB—Dementia with Lewy bodies, AD/ALB—Alzheimer’s disease/Amygdala Lewy body comorbidity, CJD/LBP—concomitant Creutzfeldt–Jakob disease and Lewy body pathology, OND—other neurodegenerative diseases, SAA—seeding amplification assay

**Fig. 6:**
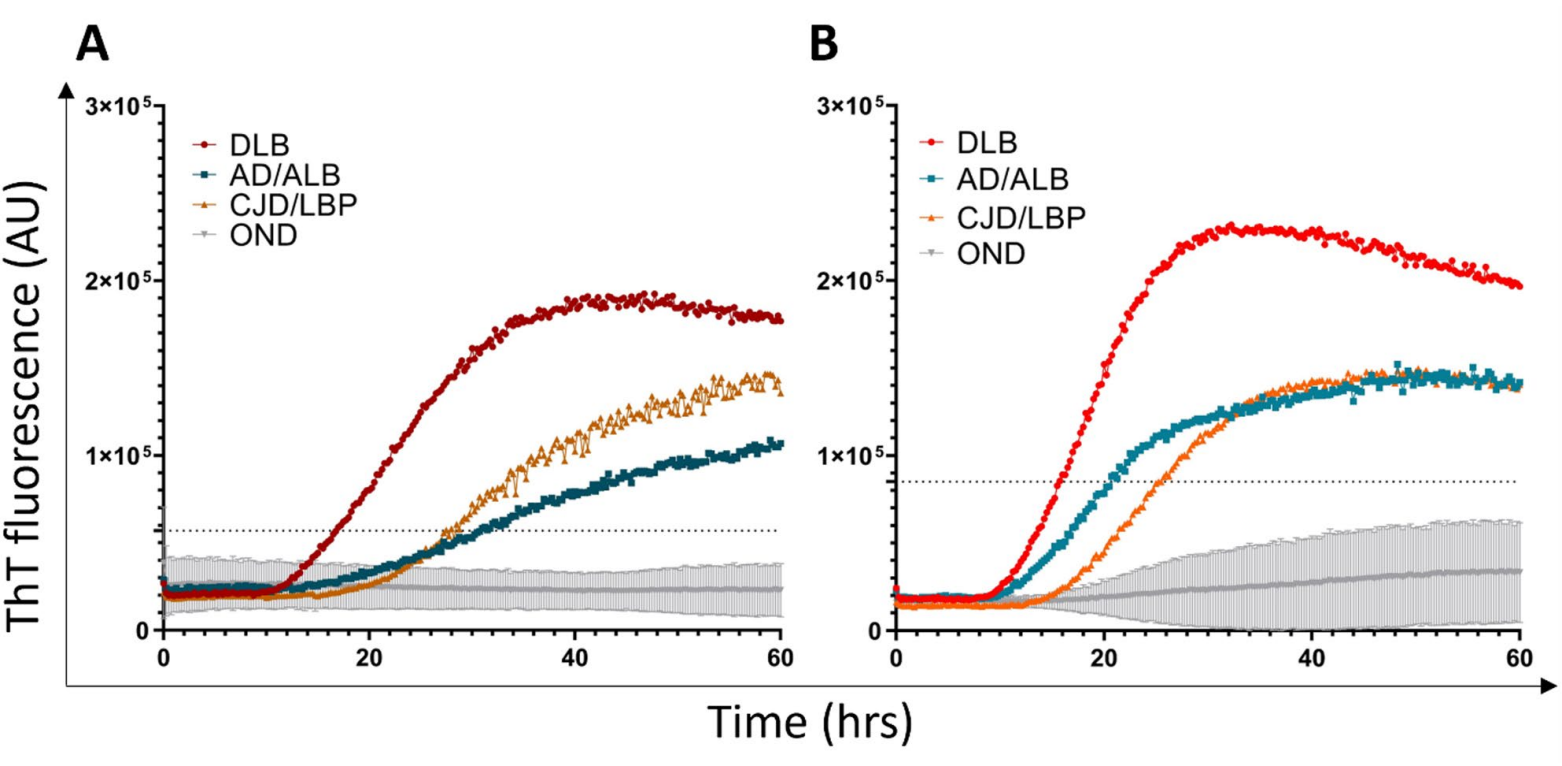
SAA analysis of α-syn^D^ seeding activity in postmortem CSF samples. The analysis of undiluted (**A**) and 10x diluted (**B**) CSF samples of DLB (n = 6, red), AD/ALB (n = 3, blue), CJD/LBP (n = 5, orange) and control OND (n = 18, grey) cases. The samples were analyzed in quadruplicates over 60 h. Each color trace represents the mean ThT fluorescence of the individual patient group at given timepoint. For the control samples, the mean ThT fluorescence ± standard deviation is presented. Dotted line represents threshold for positive results. AU—Arbitrary fluorescence unit. DLB—Dementia with Lewy bodies, AD/ALB—Alzheimer’s disease/Amygdala Lewy body comorbidity, CJD/LBP—concomitant Creutzfeldt–Jakob disease and Lewy body pathology, OND—other neurodegenerative diseases, SAA—seeding amplification assay

The analysis of patients’ undiluted *postmortem* ventricular CSF samples (n = 14) resulted in one CJD/LBP sample not reaching the threshold, indicating 92.9% (95% CI 66.1–99.8%) assay sensitivity. The mean max ThT fluorescence of DLB samples (20 ± 1 × 10^4^) was significantly higher than AD/ALB samples (11 ± 5 × 10^4^, *P* < 0.003) and the fluorescence of CJD/LBP samples was intermediary at 15.5 ± 8 × 10^4^ (Fig. 5A). Mean time to threshold for undiluted DLB CSF samples was notably shorter (16.5 ± 7 h) than for AD/ALB (31 ± 22 h) and CJD/LBP (31 ± 9 h) samples. Similarly, AUC for DLB samples was 7.3 × 10^6^, about twice as high as for AD/ALB (3.5 × 10^6^) and CJD/LBP (4.3 × 10^6^) samples (Fig. 6A).

Out of control OND CSF samples (n = 18), one (AD) provided signal above the threshold suggesting the assay specificity is 94.4% (95% CI 74.2–99.9%) (Fig. 5A). Two other OND samples (AD, hypoxic/anoxic brain injury) showed unusually high baseline signal, however without any aggregation activity and therefore were omitted from threshold calculations. The AD sample showed a fluorescence signal above SD but remained borderline negative. Immunohistochemistry reanalysis of these cases revealed no detectable α-syn^D^ deposits in the corresponding brain. The mean max ThT fluorescence of all undiluted CSF controls (n = 18) was 3.3 ± 2.3 × 10^4^. The time to threshold of the positive OND (AD) sample was 50 h. The mean AUC of all OND samples (n = 18) was 1.4 × 10^6^ (Fig. 6A).

The analysis of 10x diluted CSF samples led to negative SAA results in one AD/ALB and one CJD/LBP sample (Fig. 5B), corresponding to 85.7% (95% CI 57.2–98.2%) assay sensitivity. The mean ThT max for diluted DLB samples was 23.5 ± 4.8 × 10^4^, which was significantly higher (*P* < 0.05) compared to CJD/LBP samples (15.6 ± 6.6 × 10^4^). Mean ThT max for AD/ALB was 16 ± 12.3 × 10^4^ (Fig. 5B). The mean time to threshold, 16 ± 3 h for diluted DLB samples, was almost the same as for the undiluted samples. However, it was noticeably shorter for AD/ALB (22 ± 10 h) and for CJD/LBP (24 ± 4 hrs) comorbidities then for the corresponding undiluted samples. The mean AUC for diluted CSF samples was 9.5 × 10^6^ for DLB, 5.8 × 10^6^ for AD/ALB, and 5.4 × 10^6^ for CJD/LBP samples (Fig. 6B). One AD/ALB and four DLB diluted CSF samples have reached the detection limit of the reader, suggesting that the calculation of mean max ThT fluorescence and AUC of DLB and AD/ALB samples was likely to provide lower values.

Assay’s specificity was the same for diluted CSF samples as for undiluted, corresponding to 94.4% (95% CI 74.2–99.9%). Only one control OND sample (AD), which was also positive when tested undiluted, provided a fluorescence signal above the threshold. The mean max ThT signal of the CSF control group was 3.9 ± 3 × 10^4^ (Fig. 5B). The time to threshold for the one false-positive AD sample was 34 h. Finally, the mean AUC of all OND samples was 1.4 × 10^6^ (Fig. 6B).

### Detection of prion seeding activity in CJD/LBP samples by SAA

**Fig. 7:**
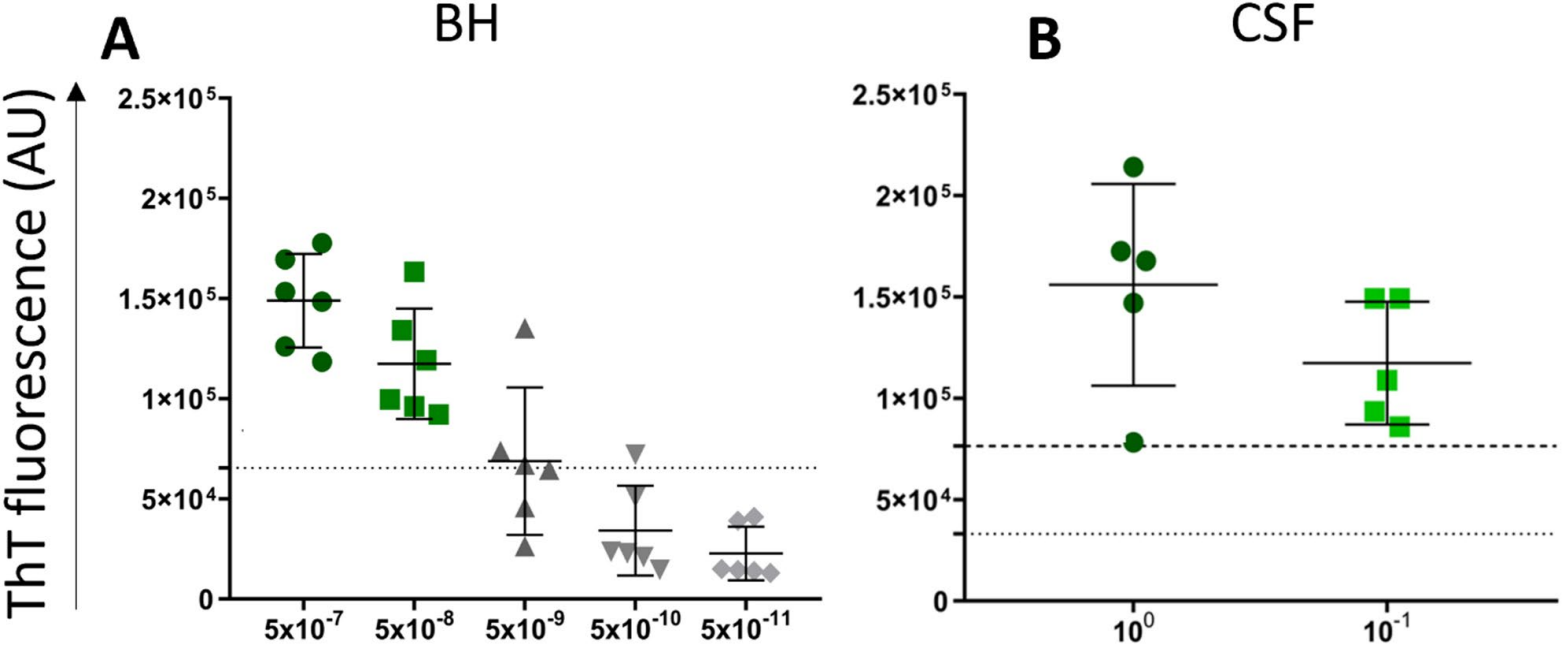
Comparison of maximal ThT fluorescence intensity of individual *postmortem* brain and CSF samples analyzed by prion SAA. The samples of CJD/LBP patients were analyzed in quadruplicates. Every dot represents the mean max ThT signal of four wells of the tested sample. Horizontal lines show the mean ± standard deviation (SD). **A** The max ThT signal of analyzed brain (n = 6) is shown for every sample in final dilutions 5 × 10^− 7^ – 5 × 10^− 11^. The threshold (dotted line) was determined for 5 × 10^-7^ dilution. **B** The max ThT signal of CSF samples (n = 5) analyzed undiluted or 10x diluted. The positivity is determined by dotted threshold for undiluted and dashed threshold for 10x diluted CSF samples. AU—Arbitrary fluorescence unit, CJD/LBP—concomitant Creutzfeldt–Jakob disease and Lewy body pathology, SAA—seeding amplification assay

**Fig. 8:**
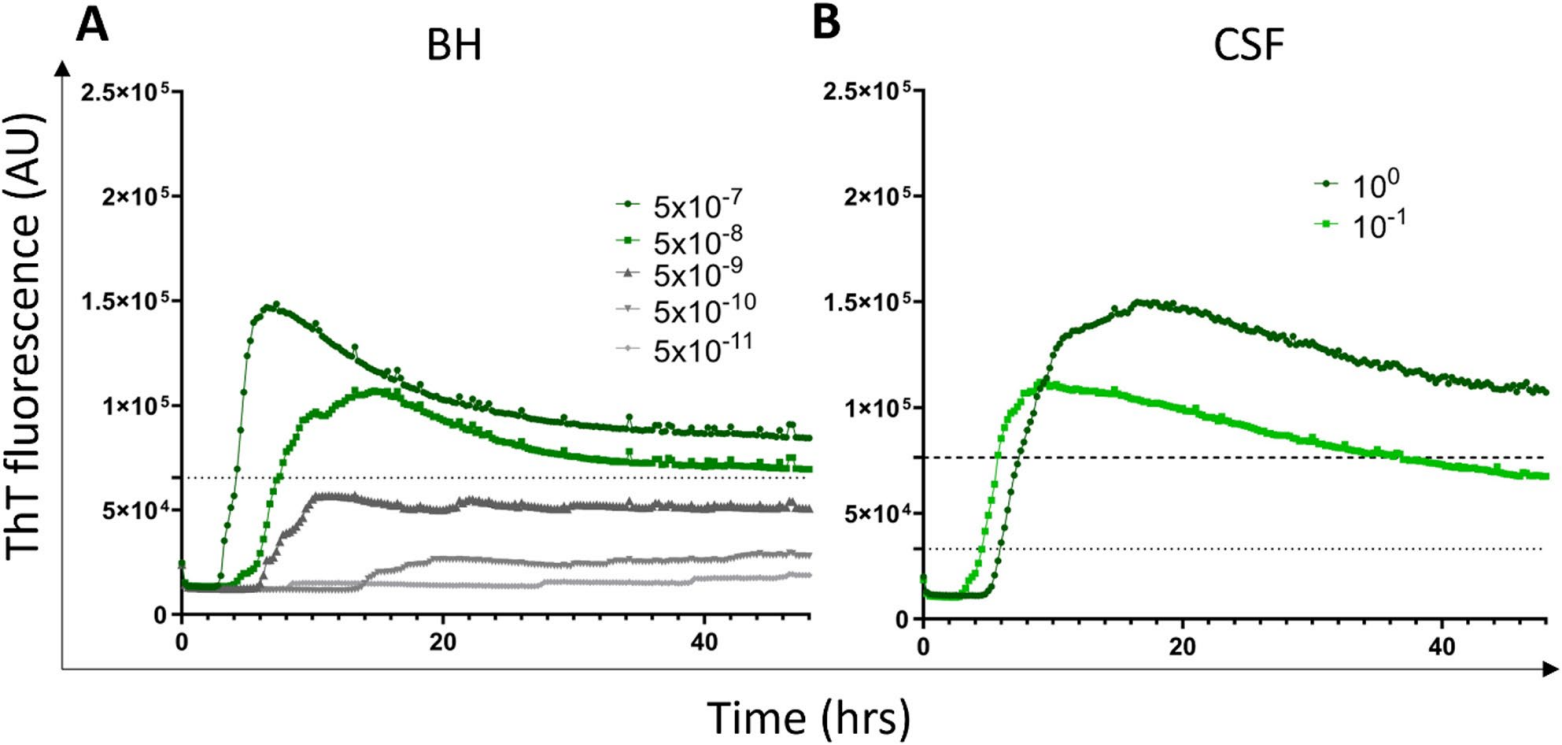
SAA analysis of prion seeding activity in postmortem brain and CSF samples of patients with CJD/LBP co-pathology. The samples were analyzed in quadruplicates. Each trace shows the mean ThT fluorescence of all tested samples during the 48 h. **A** The mean ThT fluorescence intensity of brain samples (n = 6) serially diluted from 5 × 10^− 7^ to 5 × 10^− 11^. Dotted lines represent threshold for the positive result at 5 × 10^− 7^ dilution. **B** The mean kinetics of prion seeding activity from CSF samples (n = 5) analyzed undiluted or 10x diluted. Dotted and dashed lines represent threshold calculated for undiluted and diluted samples, respectively. AU—Arbitrary fluorescence unit, CJD/LBP—concomitant Creutzfeldt–Jakob disease and Lewy body pathology, SAA—seeding amplification assay

All brain samples of CJD/LBP patients (n = 6) were classified as prion SAA positive up to 5 × 10^− 8^ dilution, suggesting 100% (95% CI 54.1–100%) assay sensitivity. At 5 × 10^− 9^ dilution, only two brains gave positive results, and at 5 × 10^− 11^ dilution, none was positive (Figs. 7A and 8A). The mean max ThT fluorescence signal at 5 × 10^− 7^ dilution was 14.9 ± 2.3 × 10^4^ (Fig. 7A).

Similarly, all undiluted and 10x diluted CSF samples (n = 5) gave positive SAA results corresponding to 100% (95% CI 56.6–100%) assay sensitivity (Figs. 7B and 8B). The mean max ThT fluorescence for undiluted and diluted CSF samples was 15.6 ± 5 × 10^4^ and 11.7 ± 3 × 10^4^, respectively. The mean SD_50_ of prions in the brain was 10^11.6^/g (Supplementary Table 5), approximately 3 orders of magnitude higher than the SD_50_ of α-syn^D^. Detailed analysis is provided in the supplemental data.

.

## Discussion

Despite many advances in diagnostics, there is still no definitive *antemortem* test for synucleinopathies in clinical use. SAA have shown promise [15] in detecting α-syn^D^ seeding activity, but variability in methods [40] and limited data from retrospective studies with definite diagnoses remain challenges. To address this, we analyzed archived *postmortem* BH and CSF from cases with primary or co-pathology synucleinopathy.

Exploiting the approach of Dr. Caughey’s lab [16], utilizing WT rec.αSyn substrate with N-terminal His-tag, we were able to confirm the positive seeding activity in all patient brain samples. We observed partial quenching of the α-syn^D^ seeding activity at the lowest dilution of 10^− 2^, leading to slower substrate aggregation likely due to the presence of SAA inhibitors in the brain tissue, as was reported previously [10]. At a 10^− 3^ dilution, the inhibitors seemed to be diluted out, and the SAA reaction proceeded successfully. In comparison, prion SAA is usually completely inhibited up to 10^− 3^ dilution of brain. However, the overall ThT fluorescence of the patient samples with synucleinopathies was relatively low and partially overlapped with the signal of control samples, which was in contrast with the other reported studies [16, 18]. To improve the discrimination of the assay for brain samples, we have included a low concentration of SDS in the reaction mix to accelerate the aggregation and N-2 supplement to prevent loses of ɑ-syn^D^ seeds at high dilutions [30]. A similar approach for brain samples was exploited by Jin et al. [22], who used 0.0006% SDS in the reaction mix. After adding 0.0005% SDS and N-2 supplement, we observed significantly increased fluorescence signal and modest shortening of the lag phase of patient brain samples. Again, partial inhibition of the reaction was observed at a 10^− 2^ dilution, but further dilution led to improved SAA kinetic. After optimization, the mean SD_50_ of brain samples was approximately ten times higher when compared to the assay without SDS. The seeding activity of one DLB sample could not be fully diluted, even up to a 10^− 8^ dilution of the brain. This observation may be attributed to advanced pathology associated with the late stage of DLB. It is important to note that because we wanted to compare two conditions of the SAA reaction, we used the same setting for the fluorescence measurement. Therefore, almost all brain samples analyzed by protocol with SDS reached the detection limit of the reader which restricted analysis of differences in fluorescence parameters among the synucleinopathy groups.

Importantly, we confirmed the presence of α-syn^D^ seeding activity in the frontal lobes of patients with AD/ALB, where α-syn^D^ deposits were not detected neither by neuropathological examination nor by immunohistochemistry using different anti-α-syn antibodies. Surprisingly, the mean time to threshold of AD/ALB and DLB samples was similar, suggesting the presence of comparable levels of α-syn^D^ seeding activity. More precise endpoint dilution of brain samples showed SD_50_ of AD/ALB frontal lobe approximately twentyfold lower than of classical DLB cases with abundant frontal lobe LB. Comparable results in patients with amygdala predominant LB pathology were reported by Bentivenga et al., where out of 138 brain samples, 47 showed positive seeding activity despite negative immunohistochemistry (IHC) staining [5]. While the nature of this observation is not known, speculation whether the seeding activity is caused by a new, different strain of ɑ-syn^D^ present in the frontal lobe or by the strain that is present in the amygdala LB has been presented [4].

Moreover, to our best knowledge, we were the first to further demonstrate both ɑ-syn and prion seeding activity in the brains of patients with CJD/LBP co-pathology utilizing distinct SAA, specifically RT-QuIC assays. This finding underscores the importance of better understanding the occurrence of co-pathologies and improving differential diagnosis in mixed dementia cases.

Analyzing control groups utilizing the same assay conditions, we could not get 100% specificity. In the OND control group, we observed higher α-syn^D^ seeding activity in three AD samples, which is consistent with other studies [5, 7, 22, 25]. After optimization of the assay, we got positive results for the same AD patients, two of which were re-evaluated as having concomitant synucleinopathy by immunohistochemistry. In the absence of a sensitive confirmatory method, we cannot rule out the possibility that this tendency was caused by the presence of low ɑ-syn^D^ seeding activity in brain tissue, as deposits of ɑ-syn^D^ can be found in AD [43]. For the CD control group, we observed two samples that were repeatedly highly positive under both assay conditions. The extended lag phase seen in both samples might be consistent with either a reduced amount of seeding activity influenced by the lower concentration of accessible seed [36] or the involvement of a different α-synuclein strain [4]. However, as the organ donors are anonymized and only BH samples were available, we could not investigate this interesting phenomenon more closely.

SAA analysis of corresponding *postmortem* ventricular CSF samples was more straightforward, and differences between synucleinopathy groups were more significant compared to brain samples. Based on previous experiences, we have analyzed CSF samples either undiluted or diluted to prevent the possibility of false-negative results caused by SAA inhibitors [28]. With this protocol, we have been able to confirm positive seeding activity in all CSF samples from patients with neuropathologically confirmed synucleinopathy.

Undiluted, DLB cases gave the highest ThT signal with the lowest dispersion of max ThT values, similarly to BH samples. Also, the initiation of aggregation was noticeably faster compared to AD/ALB and CJD/LBP samples. Comparable SAA results for DLB cases, with very high sensitivity and specificity, were reported by many others in the last years [14, 24, 35]. Moreover, we also confirmed positive seeding activity in all *postmortem* CSF samples with co-pathology ɑ-synucleinopathies. At first, one CJD/LBP sample did not reach the threshold and was classified as negative. However, the sample became positive after the dilution. Therefore, it is probable that the negative result was caused by the inhibition of seed amplification. When comparing to DLB samples, the CSF mean max ThT fluorescence was lower, specifically in AD/ALB cases, and the dispersion was higher. Compared to our results, Bongianni et al. [7] confirmed seeding activity in 14 out of 15 patients’ CSF with DLB/AD and in 2 out of 3 patients with CJD/LBP diagnosis. Also, Hall et al. [17] reported less robust SAA read-outs when testing CSF samples from patients with amygdala or brainstem restricted LB. Analyzing controls, which consisted of a distinct set of patients with OND, we observed a positive ThT signal in two samples with AD and one sample with hypoxic/anoxic brain injury. SAA positivity in *antemortem* CSF from AD patients was already observed by different groups [5, 14, 17]. Also, Verdurand et al. [42] analyzed *antemortem* CSF samples and reported six (14.3%) AD cases that tested positive in α-syn SAA, from which five were later reclassified as AD/LBP upon review of clinical records. Consistent with our findings, the same group also observed a longer lag phase in those patients.

When analyzing diluted *postmortem* CSF, the average maximal ThT fluorescence for DLB samples modestly increased; however, for AD/ALB and CJD/LBP samples, we did not observe any improvement. After the dilution, the aggregation of α-synuclein was initiated earlier, but there was bigger dispersion of max ThT fluorescence values, especially for the samples with comorbidities. Moreover, one AD/ALB and one CJD/LBP case that were positive when tested undiluted did not reach the threshold, and were classified as negative, possibly because of dilution of ɑ-syn^D^ seeds in the sample, which is in line with Mastrangelo et al. and Bernhardt et al. [6, 26]. From the OND control group, only one sample with AD with very long PMI gave a positive signal, which could be either caused by the presence of α-syn^D^ not detected by IHC or possibly by some artificial *postmortem* changes in the ventricular CSF composition during the long storage of the body before the autopsy.

In conclusion, we confirmed the seeding activity in all *postmortem* brain samples and ventricular CSF samples with either primary or co-pathology synucleinopathy. We also assessed both prion and α-syn^D^ seeding activity in individuals with comorbid CJD/LBP. However, more essentially, we observed α-syn^D^ seeding activity in the frontal lobe within AD brains with amygdala isolated LB and in four control AD cases, two of which were subsequently reclassified as AD/ALB and AD/LBP. Special attention should be given to these cases during evaluation and SAA testing, as early detection of α-syn^D^ co-pathology in primary AD may improve clinical management and prognosis, given the association with more severe cognitive decline [11]. More analyses are needed to fully understand and validate the SAA performance during early stages of synucleinopathies, especially with co-pathology development in vivo. Importantly, exploring correlations of early stages α-syn^D^ seeding activity between brain, CSF, and peripheral tissues such as skin or olfactory mucosa holds great promise for a better understanding of disease origin and development of pathology.

## Conclusion

In the present study, we have confirmed the high performance value of the SAA assay also for samples of patients with co-pathology synucleinopathies. The analysis of *postmortem* CSF has suggested higher variability of α-syn^D^ seeding activity in samples from patients with co-pathology synucleinopathies compared to samples of patients with primary DLB. An interesting finding was the presence of high α-syn^D^ seeding activity in the IHC negative frontal brain lobe tissue of patients with amygdala-isolated Lewy bodies. Moreover, in two negative control AD cases, α-synuclein co-pathology was identified only after a positive SAA outcome and subsequent IHC reanalysis of additional brain regions. This finding suggests the possible value of SAA in improving *postmortem* detection of co-pathology synucleinopathies in ongoing and future clinical studies.

## Supplementary Information

Below is the link to the electronic supplementary material.

Supplementary Material 1

Supplementary Material 2

Supplementary Material 3

## Data Availability

All SAA analyses are available at the Prion Laboratory, Institute of Medical Microbiology, First Faculty of Medicine, Charles University, Prague, Czech Republic. All data will be provided by the corresponding author upon request or can be found in the Supplement material.

## Abbreviations

AD: Alzheimer´s disease
ALB: Amygdala restricted Lewy body
ɑ-syn^D^: Pathological alpha-synuclein
AU: Arbitrary units
AUC: Area under the curve
BH: Brain homogenate
CD: Corneal donor
CJD: Creutzfeldt-Jakob disease
CSF: Cerebrospinal fluid
DLB: Dementia with Lewy bodies
FFPE: Formalin-fixed paraffin-embedded
LB: Lewy bodies
LBP: Lewy body pathology
MSA: Multiple system atrophy
OND: Other neurodegenerative diseases
PD: Parkinson´s disease
PDD: Parkinson´s disease dementia
PFF: Pre-formed fibrils
rec. HaPrP90-230: Recombinant shortened hamster prion protein
rec.αSyn: Recombinant wild-type human α-synuclein with N-terminal HisTag
RT-QuIC: Real-Time Quaking-Induced Conversion assay
SAA: Seeding amplification assay
SD: Standard deviation
SD_50_: Seeding dose of 50%
TDP-43: TAR DNA-binding protein 43
ThT: Thioflavin T
