# Supplementary material for: Alpha-synuclein seeding activity in postmortem tissues from patients with diffuse and isolated Lewy bodies
Source: Acta Neuropathol Commun. 2026 Jan 7;14:12. doi: 10.1186/s40478-025-02195-6 (PMC12781613; doi:10.1186/s40478-025-02195-6)
Supplement: Supplementary file 3 — Supplementary Material 3 [file 40478_2025_2195_MOESM3_ESM.docx]

**Supplementary table 1:** Characteristic, demographic information and SAA results of the control brain homogenates (BH) patient cohort. BH were analyzed in the absence or presence of SDS. The diagnosis of patients was confirmed by neuropathological examination during the autopsy.

| **sample** | **age** | **sex** | **PMI**  **[h]** | **definite diagnosis** | **SAA result for BH**  **with SDS** |
| --- | --- | --- | --- | --- | --- |
| 92/16 | 72 | F | 56/6 | AD A3B3C3 | **+** |
| 116/17 | 80 | F | 53/5 | AD A3B3C3 | **-** |
| 127/13 | 62 | M | NA/9 | AD A3B3C3 | **+** |
| 155/13 | 60 | M | NA/9 | AD A3B3C3 | **+** |
| 129/18 | 89 | M | NA/4 | AD A3B2C3 PSP3 | **-** |
| 119/18 | 75 | F | NA/4 | AD A3B3C3 PSP5 | **-** |
| 102/18 | 72 | M | NA/4 | AD A3B3C3 FTLD-tau | **-** |
| 25/18 | 69 | M | 24/4 | FTLD-tau PSP5 | **-** |
| 80/18 | 84 | M | 37/4 | FTLD-tau PSP6 | **-** |
| 38/18 | 64 | F | 63/4 | FTLD-tau PSP6 | **-** |
| 135/17 | 73 | F | 101/5 | FTLD-tau PSP5 | **-** |
| 93/15 | 67 | F | 31/7 | FTLD-TDP | **-** |
| 110/16 | 72 | F | 39/6 | FTLD-TDP | **-** |
| 60/19 | 66 | M | 18/3 | FTLD-TDP | **-** |
| 76/15 | 51 | M | 89/7 | FTLD-TDP | **-** |
| 121/12 | 80 | M | 80/10 | HD | **-** |
| 37/13 | 61 | F | N/A/9 | HD | **-** |

AD – Alzheimer disease, FTLD-tau – frontotemporal lobar degeneration with τ pathology, FTLD-TDP – frontotemporal lobar degeneration with TDP-43-immunoreactive pathology, HD – Huntington disease, SAA – seeding amplification assay

**Supplementary table 2:** Characteristic, demographic information and SAA results of the CSF control patient cohort. CSF was analyzed both undiluted and 10x diluted. The diagnosis of patients was confirmed by neuropathological examination during the autopsy.

| **sample** | **age** | **sex** | **PMI [h] /**  **storage time (years)** | **definite diagnosis** | **SAA result for CSF**  **10^0^/ 10^-1^** |
| --- | --- | --- | --- | --- | --- |
| 78/18 | 73 | F | 22/4 | AD A3B3C3 | -/- |
| 61/18 | 74 | M | 77/4 | AD A3B2C3 | -/- |
| 85/18 | 88 | M | 140/4 | AD A3B2C3 | +/+ |
| 83/19 | 94 | F | 30/4 | FTLD-tau + VaD | -/- |
| 38/18 | 64 | F | 63/4 | FTLD-tau PSP5 | -/- |
| 40/19 | 71 | M | 22/3 | FTLD-UPS +VaD | -/- |
| 30/19 | 75 | M | 35/3 | CJD (MM1) | -/- |
| 88/19 | 70 | F | 17/3 | CJD (MM1+2) | -/- |
| 75/19 | 63 | F | 35/3 | CJD (MM1+2) | -/- |
| 130/19 | 73 | M | 16.5/3 | CJD (MV1) | -/- |
| 13/19 | 65 | F | 66/3 | CJD (MV1) | -/- |
| 35/20 | 75 | F | 36/2 | CJD (MV1) | -/- |
| 27/19 | 87 | M | 29/3 | CJD (MM1) | -/- |
| 111/18 | 73 | M | 8/4 | CJD (VPSPr) | -/- |
| 60/20 | 73 | M | 38/2 | CJD (VV2) | -/- |
| 62/18 | 34 | M | 26/4 | H/A BI | -/- |
| 10/19 | 49 | F | 62/3 | H/A BI | -/- |
| 74/19 | 56 | M | 29.5/3 | ND-A | -/- |

AD – Alzheimer disease, FTLD-tau – frontotemporal lobar degeneration with τ pathology, VaD – vascular dementia, FTLD-UPS – frontotemporal lobar degeneration with ubiquitin proteasome system immunoreactive pathology, CJD – Creutzfeldt-Jakob disease, H/A BI – hypoxic/anoxic brain injury, ND-A – non-dementia-alcoholism, SAA – seeding amplification assay

**Pre-formed rec.αSyn amyloid fibrils**

Pre-formed amyloid fibrils (PFFs) were prepared by continuous shaking of 500 µl aliquot of rec.ɑSyn in water (1 mg/ml) at 1000 rpm for 5 days at 37 °C (Eppendorf ThermoMixer C). The formation of PFFs was confirmed by specific binding of fluorescent dye Thioflavin T (ThT) and by the transmission electron microscopy (TEM).

For ThT assay, 2.5 µl of PFFs was added into 95 µl of 25 µM ThT in PBS, pH 7.4 and gently mixed with a pipette. After 30 min incubation, fluorescence was measured at 450 ± 10 nm excitation and 480 nm emission (FLUOstar Omega reader, BMG LABTECH GmbH).

For TEM, electron microscopy grids (FCF200-Cu, Electron Microscopy Sciences) were incubated on drops of PFFs for 30 min. The grids were washed twice on a drop of mQH_2_O for 30 sec and contrasted with 1% uranyl acetate for 2 - 4 min. Uranyl acetate solution was blotted out with a filter paper, the grids air-dried and observed with JEOL 1011 electron microscope equipped with a Valeta CCD camera and Olympus Software (Olympus Soft Imaging Solution GmbH).


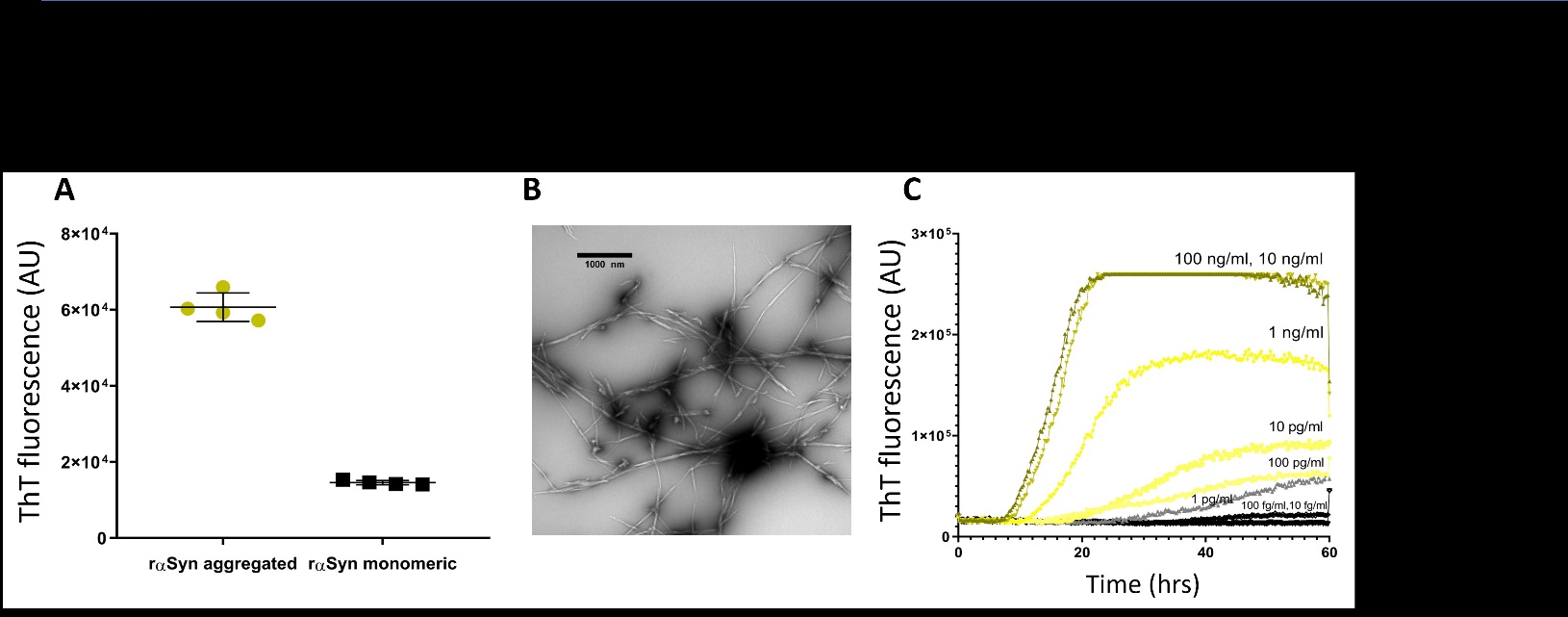


**Supplemetary Figure 1: Formation of rec.αSyn pre-formed fibrils (PFFs) and their utilization for determination of SAA assay sensitivity.** (A) ThT assay confirms the presence of the amyloid in the aggregated rec.αSyn. Monomeric rec.αSyn was used as a negative control. (B) Confirmation of PFFs' presence by TEM. The scale bar corresponds to 1000 nm. (C) Analysis of the SAA sensitivity by end-point dilution of PFFs in CSF. The serially diluted samples were analyzed in quadruplicates. Each trace represents the mean ThT fluorescence from four wells for every dilution.

AU – arbitrary fluorescence unit


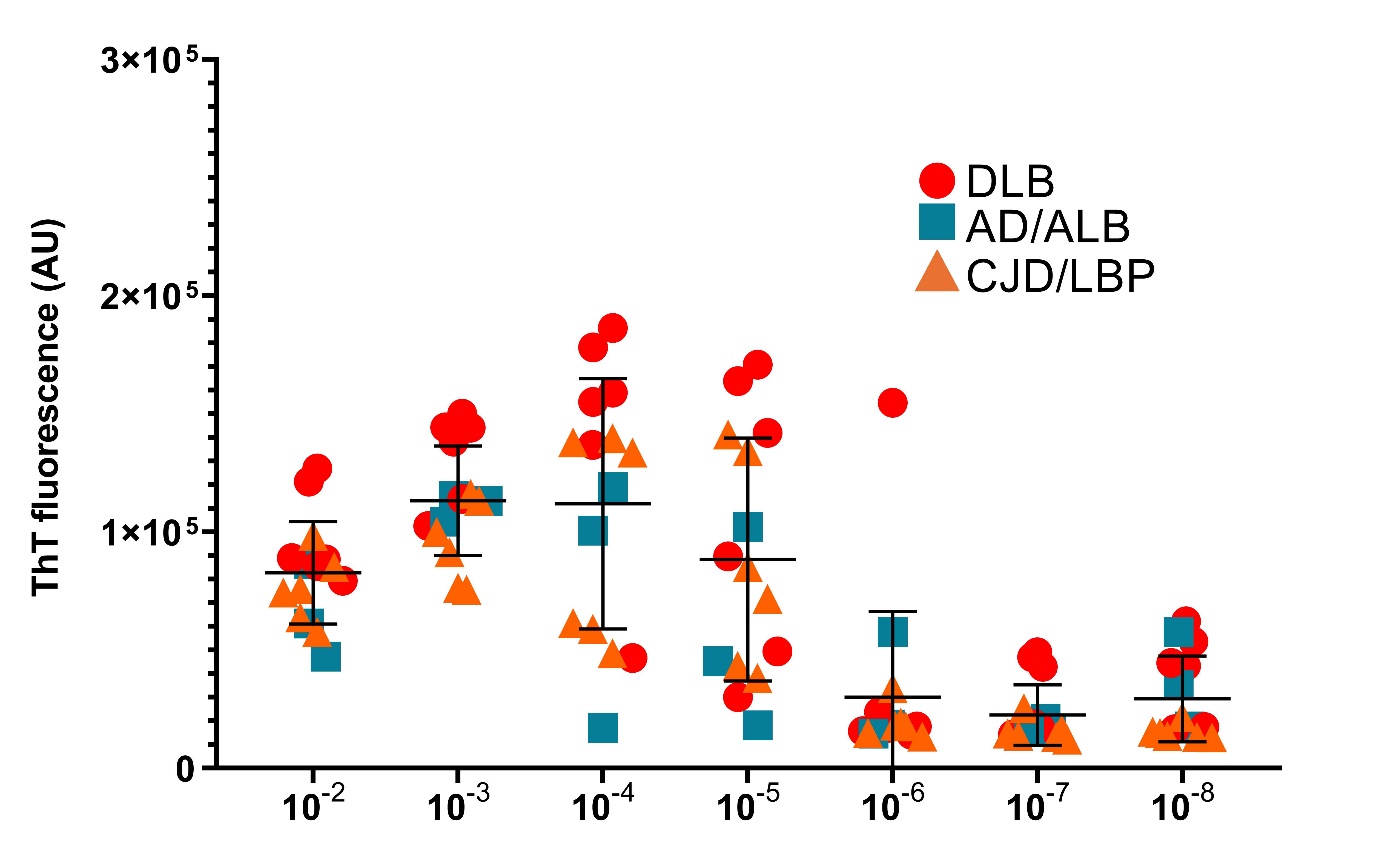


**Supplementary Figure 2: Maximal ThT fluorescence intensity of individual postmortem brain samples analyzed in end-point by α-syn SAA assay.** Samples were analyzed as quadruplicates in the absence of SDS.

DLB – dementia with Lewy bodies (n=6), AD/ALB – Alzheimer's disease/Amygdala Lewy body comorbidity (n=3), CJD/LBP – concomitant Creutzfeldt-Jakob disease and Lewy body pathology (n=6), AU – arbitrary fluorescence unit

**Supplementary table 3:** Seeding dose 50% (SD_50_) of individual brains from patients with confirmed synucleinopathies analyzed using SAA assay.

| **DLB** | | **AD/ALB** | | **CJD/LBP** | |
| --- | --- | --- | --- | --- | --- |
| **log_10_ SD_50_/**  **2 µl** | **log_10_ SD_50_/**  **1 g** | **log_10_ SD_50_/**  **2 µl** | **log_10_ SD_50_/**  **1 g** | **log_10_ SD_50_/**  **2 µl** | **log_10_ SD_50_/**  **1 g** |
| 3.75 | 6.4 | 4.25 | 6.9 | 5 | 7.7 |
| 5.5 | 8.2 | 4.75 | 7.4 | 4.5 | 7.2 |
| 4.5 | 7.2 | 3.5 | 6.2 | 4.25 | 6.9 |
| 6.75 | 9.4 |  |  | 5.5 | 8.2 |
| 5 | 7.7 |  |  | 4 | 6.7 |
| 4.75 | 7.4 |  |  | 5.5 | 8.2 |

DLB – dementia with Lewy bodies, AD/ALB – Alzheimer disease/Amygdala Lewy body comorbidity, CJD/LBP – concomitant Creutzfeldt-Jakob disease and Lewy body pathology


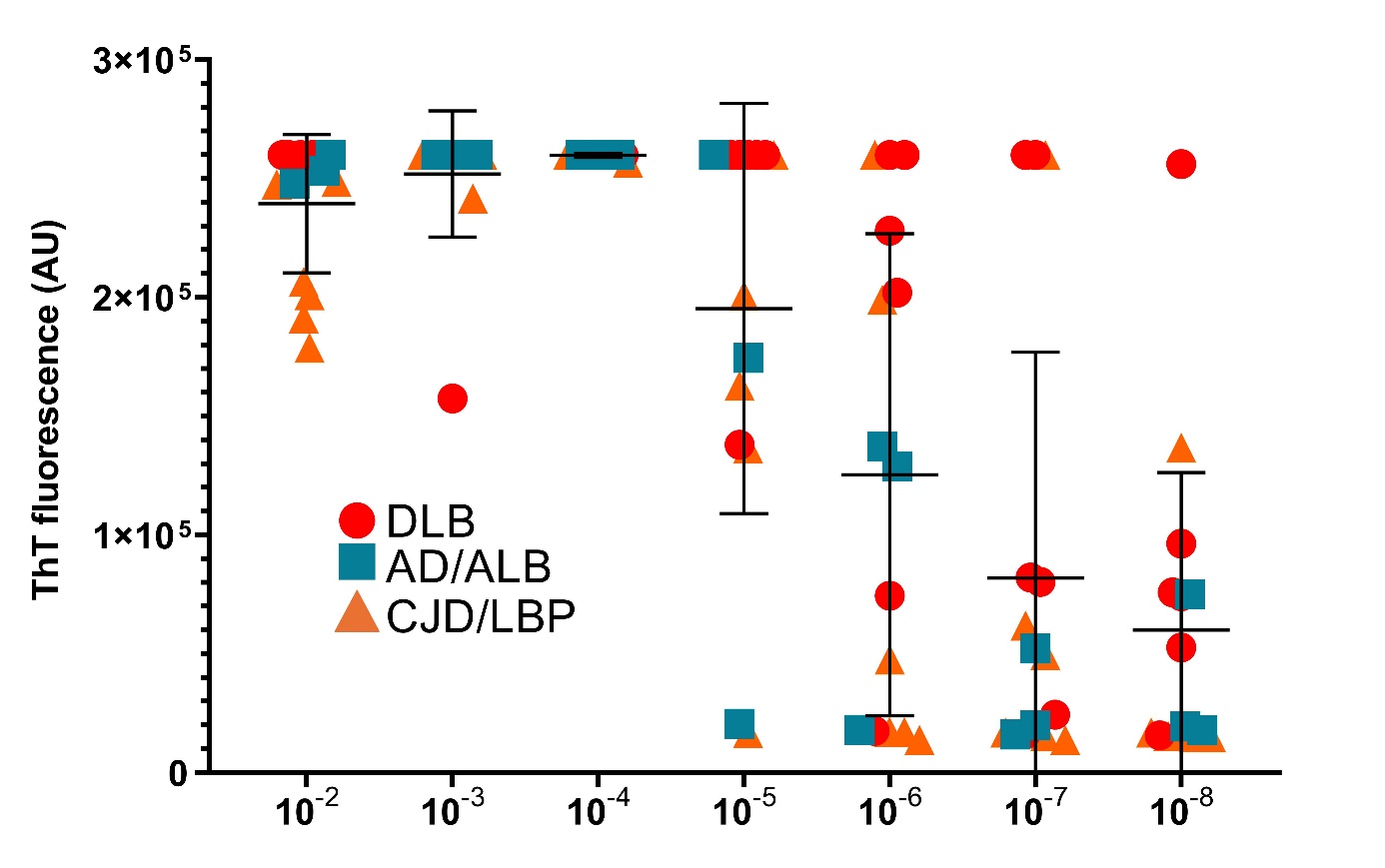


**Supplementary Figure 3: Maximal ThT fluorescence intensity of individual postmortem brain samples analyzed in end-point by α-syn SAA assay.** Samples were analyzed as quadruplicates in the presence of SDS.

DLB – dementia with Lewy bodies (n=6), AD/ALB – Alzheimer's disease/Amygdala Lewy body comorbidity (n=3), CJD/LBP – concomitant Creutzfeldt-Jakob disease and Lewy body pathology (n=6)

**Supplementary table 4:** Seeding dose 50% (SD_50_) of individual brains from patients with confirmed synucleinopathies analyzed using SAA assay with addition of 0.0005% SDS.

| **DLB** | | **AD/ALB** | | **CJD/LBP** | |
| --- | --- | --- | --- | --- | --- |
| **log_10_ SD_50_/**  **2 µl** | **log_10_ SD_50_/**  **1 g** | **log_10_ SD_50_/**  **2 µl** | **log_10_ SD_50_/**  **1 g** | **log_10_ SD_50_/**  **2 µl** | **log_10_ SD_50_/**  **1 g** |
| 5 | 7.7 | 4.5 | 7.2 | 5.25 | 7.9 |
| 7.5 | 10.2 | 6.25 | 8.9 | 5.75 | 8.4 |
| 6.5 | 9.2 | 5.25 | 7.9 | 4.5 | 7.2 |
| 8.5 | 11.2 |  |  | 6.5 | 9.2 |
| 6.5 | 9.2 |  |  | 5 | 7.7 |
| 5.75 | 8.4 |  |  | 8.5 | 11.2 |

DLB – dementia with Lewy bodies, AD/ALB – Alzheimer disease/Amygdala Lewy body comorbidity, CJD/LBP – concomitant Creutzfeldt-Jakob disease and Lewy body pathology

**Detection of prion seeding activity in CJD/LBP samples by SAA – detailed analysis**

The mean TTT was 4.3±0.7 hrs. The mean AUC for BHs was 4.6 x 10^6^. The mean TTT was 6.8±0.8 hrs for undiluted and 6.1±1.5 hrs for diluted samples. The mean AUC was 5.3 x 10^6^ for undiluted and 3.9 x 10^6^ for diluted CSF.

**Supplementary table 5:** Seeding dose 50% (SD_50_) of pathologic prion protein in brain from patients with confirmed CJD/LBP pathology analyzed using SAA assay.

|  | **log_10_ SD_50_/**  **2 µl** | **log_10_ SD_50_/**  **1 g** |
| --- | --- | --- |
|  | 8.8 | 11.5 |
|  | 9 | 11.75 |
|  | 8.3 | 11 |
|  | 8.8 | 11.5 |
|  | 10.3 | 13 |
|  | 8 | 10.75 |
| **Average** | **8.9** | **11.6** |

CJD/LBP – concomitant Creutzfeldt-Jakob disease and Lewy body pathology
